# Progress on role of ion channels of cardiac fibroblasts in fibrosis

**DOI:** 10.3389/fphys.2023.1138306

**Published:** 2023-03-09

**Authors:** Chenxv Xing, Limeng Bao, Weidong Li, Hongkun Fan

**Affiliations:** Department of Physiology and Neurobiology, School of Basic Medical Sciences, Zhengzhou University, Zhengzhou, China

**Keywords:** cardiac fibrosis, cardiac fibroblasts, ion channels, Ca^2+^, TRP channels, Piezo 1, CRAC, VGCCs, sodium channels, potassium channels

## Abstract

Cardiac fibrosis is defined as excessive deposition of extracellular matrix (ECM) in pathological conditions. Cardiac fibroblasts (CFs) activated by injury or inflammation differentiate into myofibroblasts (MFs) with secretory and contractile functions. In the fibrotic heart, MFs produce ECM which is composed mainly of collagen and is initially involved in maintaining tissue integrity. However, persistent fibrosis disrupts the coordination of excitatory contractile coupling, leading to systolic and diastolic dysfunction, and ultimately heart failure. Numerous studies have demonstrated that both voltage- and non-voltage-gated ion channels alter intracellular ion levels and cellular activity, contributing to myofibroblast proliferation, contraction, and secretory function. However, an effective treatment strategy for myocardial fibrosis has not been established. Therefore, this review describes the progress made in research related to transient receptor potential (TRP) channels, Piezo1, Ca^2+^ release-activated Ca^2+^ (CRAC) channels, voltage-gated Ca^2+^ channels (VGCCs), sodium channels, and potassium channels in myocardial fibroblasts with the aim of providing new ideas for treating myocardial fibrosis.

## 1 Introduction

There are many types of cardiac cells, among which fibroblasts account for 75% of the total cells. Cardiac fibroblasts (CFs) produce extracellular matrix (ECM) by secreting collagen, and its imbalanced synthesis and metabolism will lead to excessive deposition of myocardial interstitial collagen, thereby resulting in cardiac fibrosis. Cardiac fibrosis is sometimes a restorative collagen-based scar that replaces dead cardiomyocytes to play a role in maintaining the structural integrity of chambers. Nevertheless, fibrosis destroys the coordination of myocardial excitation-contraction coupling during the systolic and diastolic period, causing serious damage to systolic and diastolic functions. In addition, the accumulation of cardiac interstitial collagen fibers will increase myocardial stiffness, resulting in reduced cardiac compliance and diastolic dysfunction. Moreover, the continuous increase can lead to ventricular dilatation, cardiac hypertrophy and congestive heart failure (HF). Therefore, the prevention and treatment of cardiac fibrosis are of great significance to the prognosis of cardiovascular diseases, but effective targets are still lacking in clinical practice. Recent studies have suggested that some ion channels in fibroblasts are related to fibroblast differentiation and cardiac fibrosis. This review summarizes the research progress of ion channels of cardiac fibroblasts in fibrosis in recent years, in order to provide new ideas for clinical treatment of myocardial fibrosis.

## 2 Correlation between fibroblasts and cardiac fibrosis

### 2.1 Cardiac fibroblasts

There are a variety of cells in the heart, they contain cardiomyocytes, fibroblasts, endothelial cells and smooth muscle cells, of which fibroblasts are the most, accounting for about 75% (Eghbali, 1992; Camelliti et al., 2005; Nemoto et al., 2022). Their main functions are to produce and maintain the homeostasis of myocardial ECM (Eghbali, 1992; Ottaviano and Yee, 2011), as well as produce growth factors (GFs), cytokines and intercellular signaling molecules, affecting cell proliferation, angiogenesis, cardiomyocyte hypertrophy and apoptosis (Ottaviano and Yee, 2011; Mitrokhin et al., 2019). Due to high impedance, the fibroblast membrane can separate the atriums and ventricles by forming fibrous rings to ensure orderly contraction of the heart (Eghbali, 1992). Myocardial fibroblasts are coupled with cardiomyocytes through gap junctions and maintain electrical conduction and communication with cardiomyocytes through gap junction proteins (Ottaviano and Yee, 2011; Mitrokhin et al., 2019). An optogenetic study verified *in vivo* that fibroblasts depolarize when coupled cardiomyocytes generate action potentials (Krishnan et al., 2021). Furthermore, fibroblasts can also directionally differentiate into other cells, such as myofibroblasts (MFs), pluripotent stem cells and neurons (Nakagawa et al., 2008). Therefore, fibroblasts play an important role in maintaining the structure, function, biochemistry and bioelectricity of the heart.

The main function of fibroblasts is to synthesize structural proteins, including ECM proteins, and maintain the homeostasis of ECM through the synthesis and degradation of connective tissue components (Frangogiannis, 2021; Frangogiannis, 2022). Cardiac ECM is mainly composed of fibrillar collagen types I and III, and a small amount of collagen IV, collagen V and collagen VI. ECM reconstruction is the key to cardiac reconstruction. Cardiac injury induces the hyperplasia of fibroblasts and trigger them to proliferate and differentiate into ECM-secreting MFs, which have a strong ability to produce ECM proteins and cause collagen accumulation, namely, myocardial fibrosis (Nakagawa et al., 2008; Liu et al., 2022). MFs are fibroblasts with transformed phenotype and the characteristics of smooth muscle (Yu et al., 2018). α-smooth muscle actin (α-SMA) is only expressed in MFs, while in non-transformed fibroblasts, there are only stress fibers composed of cytoplasmic actin, but no α-SMA expression. Consequently, α-SMA can be used as a marker to identify differentiated MFs in injured tissues (Marrouche et al., 2014; Alex and Frangogiannis, 2018). Additionally, MFs can secrete bioactive molecules, such as interleukin-1α (IL-1α), IL-1β, IL-6, IL-10 and Tumor-necrosis factor-α (TNF-α) and other inflammatory cytokines, to maintain the inflammatory response of the myocardium to injury (Nakagawa et al., 2008; Gourdie et al., 2016). During injury healing, MFs reconstruct the injured tissue by producing massive collagen to replace lost cardiomyocytes, which has the significance of repairing the injury. Regardless of the cause of cardiac injury, MFs participate in tissue repair and fibrosis by secreting matrix proteins. However, inactive cardiac fibroblasts have no α-SMA -related cytoskeleton and do not massively secrete matrix proteins (Ammarguellat et al., 2001; Borer et al., 2002; Rohr, 2012). After a cardiac injury, the changes in the matrix environment, the induction and release of GFs and cytokines, as well as the increase of mechanical stress can dynamically regulate the phenotype of fibroblasts (Berk et al., 2007; Bansal et al., 2017). Therefore, MFs transdifferentiation is a marker of fibrosis in any cardiac pathological condition.

### 2.2 Characteristics of myocardial fibrosis

Adult mammalian ventricular muscle is composed of tightly coupled cardiomyocyte layers and a highly intercellular matrix defined by a complex ECM protein network. Cardiac ECM is mainly composed of fibrillar collagen, which is not only the scaffold of cell elements but also important for the transmission of contractile force. In normal adult mammalian hearts, the thick fibers that mainly provide tensile strength for the heart are type I collagen, accounting for about 85% of the total collagen in the myocardium. In contrast, type III collagen that forms thin fibers and maintains the elasticity of cardiac ECM accounts for about 10% (Weber, 1989). Cardiac ECM also contains glycosaminoglycans, glycoproteins and proteoglycans, as well as stored potential GFs and proteases, which can be activated when damage triggers a repair response. From the perspective of morphology, cardiac ECM can be divided into epimysium, perimysium and intramuscular components (Weber, 1989). The epimysium is located on the endocardial and epicardial surface, providing support for endothelial cells and mesothelial cells. In the perimysium, the muscle fibers are arranged in bundles, while the intramuscular components are produced around the muscle and surround a single cardiomyocyte (Berk et al., 2007).

Cardiac fibrosis is a common pathological feature of most myocardial diseases. The heart of adult mammals has little regenerative capacity, and massive loss of cardiomyocytes will result in reparative fibrosis in the myocardial interstitium, which is essential for preserving the heart’s structural integrity (Travers et al., 2016). Cardiac fibroblasts are the core factor of normal cardiac physiology and cardiovascular diseases and play an important role in development by depositing collagen and other ECM components (Meagher et al., 2020). Fibroblasts constantly change the microenvironment (Mahoney et al., 2016), regulate the balance between ECM degradation and formation, and maintain the homeostasis of the internal environment by degrading and depositing ECM. However, stimulated by many factors, this balance is destroyed, and ECM formation exceeds degradation, resulting in interstitial fibrosis (Hinz, 2007; Hinz, 2010; Kong et al., 2014). Cardiac injury is usually related to the destruction of myocardial structural integrity, and cardiac fibroblasts may significantly promote the transdifferentiation of MFs due to increased mechanical stress (Nakagawa et al., 2008), so the increase in fibrous collagen accumulation in cardiac interstitium is a sign of cardiac fibrosis (Humeres et al., 2016). Regardless of the cause of fibrotic remodeling, the increased ECM is mainly fibrillar collagen I and III, and a small amount of collagen IV, collagen V and collagen VI (Alex and Frangogiannis, 2018). In the model of hypertension and myocardial infarction (MI) (Park et al., 2018), activated MFs are the main source of collagen in the fibrotic heart (Borer et al., 2002), and the upregulation of type I collagen in MFs is more prominent than type III collagen.

### 2.3 Histopathological types of myocardial fibrosis

Based on histopathological criteria, cardiac fibrosis can be divided into three different types. Replacement fibrosis reflects the formation of fibrous scar tissue in the necrotic area of cardiomyocytes, which is the main pathology of MI (Frangogiannis, 2015; Hinderer and Schenke-Layland, 2019). “Interstitial fibrosis” is used to describe intramuscular and perimuscular enlargement caused by the deposition of structural ECM proteins (Frangogiannis, 2021). The expansion of the collagen area in the adventitia of cardiac microvessels is called “perivascular fibrosis.” These differences have pathophysiological characteristics and functional significance. Replacement fibrosis is the final result of the repair response to primary necrosis of cardiomyocytes, which is usually related to systolic ventricular dysfunction. On the contrary, other nociceptive stimuli (such as mechanical stress caused by pressure overload, myocarditis, or metabolic disorders) may cause overproduction of ECM, resulting in changes in perivascular and/or interstitial fibrosis (Swynghedauw, 1999). These fibrous changes are generally related to diastolic dysfunction (Frangogiannis et al., 2002; Frangogiannis, 2015). In muscle mechanics, increased viscoelastic load, rate of relaxation, diastolic suction, and passive stiffness are due to myocardial fibrosis (Burlew and Weber, 2002; Hulsmans et al., 2018). Additionally, the increased myocardial stiffness hampers the subsequent passive inflow and causes a backward diastolic flow that further impairs early filling, resulting in a less efficient heart in the total diastolic phase (Hulsmans et al., 2018; López et al., 2021).

## 3 Activation and differentiation of fibroblasts

MFs are rarely seen in normal heart tissue. Cardiac fibroblasts which are very sensitive to stimulation always differentiate into MFs in response to tissue damage or increased mechanical stress, and increase the synthesis of ECM proteins, thus leading to myocardial fibrosis. In many cardiac injury models, neurohumoral mediators, cytokines and GFs are released and activated after myocardial injury and bind to cell surface receptors to transduce fibrogenic intracellular signal cascades. Additionally, specialized matrix proteins and matrix macromolecules enrich the matrix network, and locally activate fibroblast growth factor at the injury site, providing the spatial regulation of fibroblast activation signals (Luther et al., 2012; Skrbic et al., 2015; Goins et al., 2022). In addition to fibroblasts, several other types of cells participate in myocardial fibrosis by changing the microenvironment and affecting the phenotype of fibroblasts. Immune cells, including macrophages, mast cells and lymphocytes, are recruited and activated during cardiac remodeling and may play an important role in fibroblast activation by secreting various fibrogenic mediators (including cytokines, GFs and matricellular proteins) (Saxena et al., 2014; Nevers et al., 2015; Shiraishi et al., 2016; Carlson et al., 2017; Lafuse et al., 2020; Okyere and Tilley, 2020; Kubota and Frangogiannis, 2022). Endothelial cells, pericytes and vascular smooth muscle cells may also regulate fibroblast differentiation by secreting molecular signals that regulate fibroblast behaviors (Sun et al., 2020). When myocardial damage occurs, the death of cardiomyocytes will trigger an inflammatory response. Cardiomyocytes can produce and secrete a variety of fibrogenic media, leading to the activation of CFs and the formation of fibrous tissue to replace dead cardiomyocytes (Frangogiannis, 2008; López et al., 2021). In the mouse model, deoxycorticosterone/salt is found to mediate cardiac fibrotic remodeling, and cardiomyocyte-specific mineralocorticoid receptor signal transduction promotes cardiac fibrosis (Rickard et al., 2012). In addition, it is also found that tissue growth factor beta (TGF-β) receptor II signal transduction in cardiomyocytes plays an important role during the development of cardiac fibrosis in the model of cardiac pressure overload (Koitabashi et al., 2011; Ock et al., 2016). In infarcted myocardium, the early stimulation that triggers an inflammatory response and drives cardiac fibrosis is mediated by the release of Danger-associated molecular pattern (DAMP) by injured cardiomyocytes (Prabhu and Frangogiannis, 2016; Hu et al., 2022). Moreover, it has been proposed that TGF-β synthesis can be attenuated by the expression of plasminogen activator inhibitor-1 (PAI-1) in cardiomyocytes to protect the myocardium from fibrotic remodeling (Prabhu and Frangogiannis, 2016). However, it is unclear whether this effect is regulated by the expression profile of fibrogenic mediators in cardiomyocytes, or only reflects cardiomyocyte death.

## 4 Effects of ion channels on fibroblast differentiation or myocardial fibrosis

Although the ion channels in cardiomyocytes and their functions have been widely studied, their expressions and physiological roles in fibroblasts are still unclear. Though recognized as non-excitable cells because of the absence of an action potential on isolated cells, fibroblasts have been reported to be able to functionally express a wide variety of ion channels whose activity may contribute to the regulation of key cardiac pathophysiological processes (Li et al., 2009; Chacar et al., 2017). A resting membrane potential (RMP) of about −13 to −18 mV was determined *in situ* in cardiac atrial fibroblasts using standard sharp microelectrode recordings (Kamkin et al., 1999).Values between −34 and −40 mV have been recorded *in situ* with a patch-clamp in isolated rat atrial fibroblasts (Kamkin et al., 2003). In the fibroblasts of the canine model of HF, the RMP was hyperpolarized, from −43 mV in the control group to −56 mV in fibroblasts with HF, reflecting that the ion channels in fibroblasts were regulated by some abstruse factors and changed under pathological conditions (Chilton et al., 2005; Shibukawa et al., 2005).

### 4.1 Effect of calcium channel-mediated Ca^2+^ on cardiac fibrosis

Cardiac fibrosis is a cascade reaction in which fibroblasts sproliferate and differentiate into MFs, causing excessive deposition of matrix proteins, and many signaling pathways affect fiber production. As the secondary messenger in cells, Ca^2+^ plays an essential role in intracellular functions, including cell signal transduction, gene expression, cell proliferation, differentiation, migration, growth and death (Berridge, 2002). Although it is clear that Ca^2+^ signaling plays a vital role in the pathological activation of fibroblasts, due to the large and diverse number of receptors, transporters, and ion channels that affect intracellular Ca^2+^ kinetics, the key elements of fibroblast transdifferentiation to myofibroblasts have not been fully elucidated (Čendula et al., 2021). Decreased calcium influx into cells reduces fibroblast differentiation, collagen release, and reduce TGF-β-induced fibrotic remodeling (Ramires et al., 1998; Teng et al., 2015). Intracellular Ca^2+^ signals are generated by Ca^2+^ entry through Ca^2+^-permeable channels in the plasma membrane and by Ca^2+^ release from intracellular Ca^2+^ stores (Feng et al., 2019; Maione et al., 2022; Streiff and Sachse, 2022). Intracellular Ca^2+^ level is also precisely controlled by plasma membrane ATPase (PMCAs), Na^+^/Ca^2+^ exchangers (NCX) and sarcoplasmic reticulum Ca^2+^-ATPase (SERCA) (Kranias and Hajjar, 2012; Morotti et al., 2021). The mechanism of Ca^2+^ signaling in cardiomyocytes and its electrophysiological characteristics have been deeply understood, but the understanding of Ca^2+^ signaling in myocardial fibroblasts is limited. In recent years, a large number of studies have shown that Ca^2+^ signaling also plays a role in fibroblast physiology and fibrosis-related heart diseases (Eder, 2017).

#### 4.1.1 Transient receptor potential (TRP) channels

**FIGURE 1:**
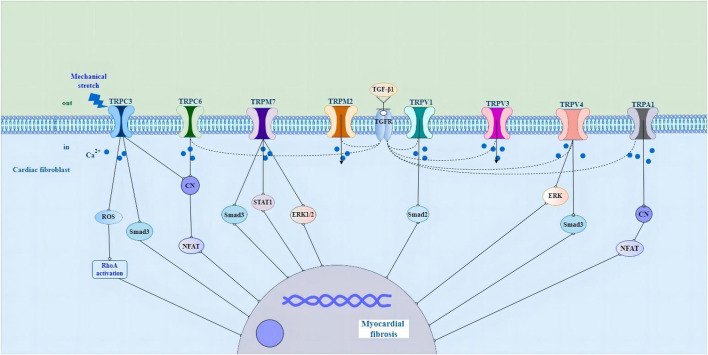
Schematic representation of the potential molecular mechanisms of TRP channels in cardiac fibroblasts in the pathological process of myocardial fibrosis. Abbreviations; TRP, transient receptor potential; ROS, reactive oxygen species; RhoA, Rho-associated kinase; CN, calcineurin; NFAT, nuclear factor of activated T cells; STAT1, signal transducer and activator of transcription1; ERK1/2, extracellular signal-regulated kinase1/2; TGF-β1, growth factor beta1; TGFR, growth factor receptor; ERK, extracellular signal-regulated kinase; See text for details.

As a non-selective cation channel with Ca^2+^ permeability, the TRP channel can not only directly initiate the cellular Ca^2+^ signal but also cause membrane depolarization and then the opening of voltage-gated Ca^2+^ channel, thus affecting Ca^2+^ influx in this indirect way (Adapala et al., 2013; Feng et al., 2019). A variety of TRP channels have been detected in mammalian CFs, such as TRPC3, TRPC6, TRPV1, TRPV2,TRPV3, TRPV4, TRPM7 and TRPA1 (Figure 1) (Du et al., 2010; Davis et al., 2012; Adapala et al., 2013; Falcón et al., 2019; He et al., 2019; Li et al., 2019; Feng et al., 2021; Hu et al., 2021; Miller et al., 2021; Oguri et al., 2021). TRPC1, TRPC4, TRPC6, TRPV2, TRPV4, and TRPM7 are expressed in cultured human cardiac fibroblasts (HCFs) (Chen et al., 2010; Du et al., 2010). In addition, TRP channels do not have a typical voltage sensor but instead can sense a variety of other stimuli, including that of mechanical stretch, pressure, shear stress, oxidative stress, alterations in the lipid environment, hypertrophic signals, and inflammatory products (Yue et al., 2015). TRP channels integrate and transduce their activity to the downstream cellular amplification system *via* Ca^2+^ entry and membrane depolarization, thereby playing a crucial role in the regulation of fundamental cellular functions such as contraction, relaxation, proliferation, differentiation, and cell death (Yue et al., 2015). Their ability to respond to various stimulating cues makes TRP channels effective sensors of the many different pathophysiological events that stimulate cardiac fibrogenesis (Feng et al., 2019).

There is evidence that the activity and expression of numerous TRP channels are upregulated in pathological myocardial hypertrophy, arrhythmia and HF (Ikeda et al., 2013; Freichel et al., 2017; Liu et al., 2018; He et al., 2019; Li et al., 2019; Saliba et al., 2019; Ahn et al., 2020; Feng et al., 2021). TRPC3 channels have been shown to play a role in cardiac fibrosis and fibrosis-associated heart diseases, such as atrial fibrillation (AF) and HF induced by pressure overload (Kiyonaka et al., 2009; Harada et al., 2012; Numaga-Tomita et al., 2017). Pharmacological inhibition of TRPC3 suppresses the fibrotic response in human cardiac myocytes and fibroblasts (Numaga-Tomita et al., 2016). TRPC3 upregulation linked to calcineurin/nuclear factor of activated T cells (NFAT) signaling in pressure-overload hypertrophy and cardiomyopathy in rodent models (Iwata et al., 2020). In cardiac hypertrophy caused by mechanical stress, the activation of TRPC3 leads to a significant increase in reactive oxygen species (ROS) production, and the activation of Rho-associated kinase (RhoA) in cardiomyocytes and fibroblasts, resulting in interstitial fibrosis, while the deletion of Trpc3 gene has an inhibitory effect on myocardial fibrosis (Kitajima et al., 2016). In neonatal rat atrial fibroblasts, Ang II induced atrial fibroblast migration and proliferation and upregulated the expression levels of TRPC3. Knocking down TRPC3 using short hairpin RNA (shTRPC3) attenuated Ang II-induced upregulation of TGF-β1 (He et al., 2019). In cardiac myocytes, the TRPC3 blocker Pyrazole-3 (Pyr3), the combined TRPC3 and TRPC6 channel blockers GSK2332255B and GSK2833503A, and the TRPC6 channel blocker BI-749327 are effective at attenuating pathological remodeling and enhancing heart function (Kiyonaka et al., 2009; Seo et al., 2014; Lin et al., 2019). TGF-β1-mediated upregulation of α-SMA in the human right ventricle was significantly reduced when TRPC6 was silenced using siRNA (Kapur et al., 2014). Moreover, treatment of the TRPC6 antagonist (BI-749327) to mice under pressure overload suppressed profibrotic gene expression, reduced cardiac fibrosis, and improved heart function (Lin et al., 2019). Activation of TRPC6 stimulates the calcineurin/NFAT pathway to induce fibroblast differentiation. Fibroblasts lacking TRPC6 (TRPC6^−/−^) were not able to differentiate into myofibroblasts in response to TGF-β1 stimulation (Davis et al., 2012). Also, it was reported that myocardial hypertrophy and fibrosis are associated with TRPC6/Calcineurin/NFAT signaling in spontaneously hypertensive rats with 5/6 nephrectomy (Bogdanova et al., 2021).

TRPV channels also play an important role in fibroblast proliferation and differentiation, and knockout of TRPV1 significantly increases myocardial fibrosis and infarction by stimulating TGF-β1 and Smad2 signaling pathways (Huang et al., 2010; Wang et al., 2014). Zhong et al. (2018) found that genetic ablation of TRPV1 channels resulted in an increased deposition of collagen in experimental hearts. Another study showed that capsaicin inhibits pressure overload-induced hypertrophy and fibrosis by activating TRPV1 (Wang et al., 2014). The activation of TRPV1 has also been shown to be protective in a MI model (Wang and Wang, 2005; Sexton et al., 2007; Zhong and Wang, 2007). In striking contrast, TRPV1 has been found to be upregulated in transverse aortic constriction (TAC)-induced pressure overload (Szabados et al., 2020). Administration of the TRPV1 antagonist BCTC (4-(3-Chloro-2-pyridinyl)-N-[4-(1,1-dimethylethyl)pheny]-1-piperazinecarboxamide) prevents loss of cardiac function in the heart (Horton et al., 2013). The cause of these discrepancies in different studies regarding the role of TRPV1 in fibrogenesis is unclear, but it could be due to the expression of TRPV1 in different cell types and the channel’s involvement in different signaling pathways (Feng et al., 2019). Liu and coworkers have shown that the activation of TRPV3 promotes the proliferation of cardiac fibroblasts through TGF-β1 pathway (Liu et al., 2018). In a rat model of pressure overload hypertrophy, carvacrol activation of TRPV3 exacerbates heart function and increases fibrosis (Liu et al., 2018; Zhang et al., 2018). Research has shown that TRPV4 regulates the differentiation of fibroblasts into myofibroblasts by integrating TGF-β1 signals and mechanical stimulation (Adapala et al., 2013; Thodeti et al., 2013). TGF-β1-induced myofibroblast differentiation is suppressed by the application of the TRPV4 antagonist AB159908 and a TRPV4 shRNA knockdown (Adapala et al., 2013). In a MI model, TRPV4 deletion in mice also decreases fibrosis and protects the heart from pathological remodeling (Adapala et al., 2017). Jia et al. (2020) revealed that TRPV4 is responsible for the activation of TGF- β 1/Smad3 signal pathway in diabetic myocardial fibrosis. TRPV4-mediated Ca^2+^ influx regulates the differentiation of human ventricular fibroblast into MFs through extracellular signal-regulated kinase (ERK) pathway (Ahn et al., 2020).

Several TRPM channels have been shown to play crucial pathophysiological roles in the heart (Takahashi et al., 2012; Rios et al., 2020; Simard et al., 2020). Takahashi et al. (2012) reported that clotrimazole-sensitive TRPM2-like currents were significantly larger in hypoxia-treated fibroblasts. In addition, TRPM2 in CFs were upregulated in AF patients in comparison with control patients (Yue et al., 2013). Research shows that the experimental strategy of treating human or animal cardiac fibroblasts with profibrogenic agonists or hormones such as angiotensin II (Ang II) significantly increases the expression of TRPM7 (Du et al., 2010). TRPM7-mediated Ca^2+^ entry contributes to enhanced Ca^2+^ influx in AF fibroblasts (Du et al., 2010). The mouse model of overexpression of TRPM7 has obvious fibrogenic effect, and this effect is transduced through Smad3 and signal transducer and activator of transcription 1 (STAT1) signal pathway (Rios et al., 2020). In primary cultured fibroblasts, oxidative stress induces the increase of fibrotic α-SMA and type I collagen by extracellular Ca^2+^ and TRPM7-mediated Ca^2+^ influx, which is accompanied by increased phosphorylation of ERK1/2 (Ahn et al., 2020). Besides, after incubating rat cardiac fibroblasts with angiotensin II, the expression level of TRPM7 protein, collagen I and III increase, promoting the occurrence of fibrosis, while the knockdown of TRPM7 protein can inhibit the differentiation of fibroblasts and the occurrence of fibrosis (Zhou et al., 2015; Li et al., 2017). TRPA1 is expressed in CFs (Li et al., 2019). Knocking down TRPA1 reduces fibrosis and enhances heart performance after MI (Li et al., 2019). Overexpression of TRPA1 in primary cultured cardiac fibroblasts enhances TGF-β1-induced fibroblast differentiation (Wang et al., 2018; Hof et al., 2019). These results suggest that TRP channels play a role in the differentiation and fibrosis of myocardial fibroblasts by regulating intracellular Ca^2+^ homeostasis, and are considered to be promising therapeutic targets for regulating pathological cardiac plasticity. However, defining the potential role of each ion channel in CF function remains challenging and needs to be carefully elucidated under various pathophysiological conditions.

In furthermore, it should be mentioned that the concept of TRP channels acting as the primary sensors of mechano-stimuli seems to be very debated (Peyronnet et al., 2016). Recent studies suggest that TRP channels may not function as direct sensors of mechano-stimuli, but rather act downstream of other primary sensors of mechanical stimulation. This seems to be despite the fact that sufficient evidence supports their role in cellular responses to mechano-stimuli (Gottlieb et al., 2008; Liu and Montell, 2015; Servin-Vences et al., 2017; Nikolaev et al., 2019). It should also be mentioned that large-conductance Ca^2+^ and voltage-activated potassium (BKCa) channels have been identified as being crucial to the biology of cardiac fibroblasts and may also respond to membrane stretch (Sheng et al., 2013). Expression of stretch-activated BKCa channels has been detected at low levels in atrial fibroblasts, however, patch clamp measurements of the stretch-induced currents in these cells were found to be largely dependent on Piezo1 activity (Jakob et al., 2021).

#### 4.1.2 Piezo1 channel

Piezo channels are mainly permeable to calcium ions and produce a non-selective flow of positively charged ions in response to mechanical stress (Li et al., 2014; Gnanasambandam et al., 2015; Zeng et al., 2018). Piezo1 and Piezo2 have similar properties, but the Piezo2 channel is activated by a higher pressure compared to Piezo1 (Volkers et al., 2015; Wu et al., 2017; Wang et al., 2019; Du et al., 2020; Szczot et al., 2021). Piezo1 is a calcium-permeable ion channel that displays synergistic or opposing functions depending on the cell type in which it is expressed (Volkers et al., 2015; Barbeau et al., 2021). Cardiac-specific knockdown or overexpression of Piezo1 in mice both resulted in defective Ca^2+^ and ROS signaling, as well as cardiomyopathy (Douguet et al., 2019; Jiang et al., 2021a). A study by Zhang et al. showed that Piezo1 expression is increased during fibrosis in cardiac hypertrophy (Zhang et al., 2021). Upon mechanical or agonist stimulation, Piezo1 evokes Ca^2+^-mediated calpain and calcineurin activation, and fibroblasts develop into myofibroblasts (Beech and Kalli, 2019; Jiang et al., 2021b; Zhang et al., 2021). In animal and human samples, Piezo1 is expressed at higher levels in fibroblasts than in cardiomyocytes (Alharbi et al., 2022). Piezo1 is associated with altered gene expression affecting CFs remodeling (Beech and Kalli, 2019). Blythe et al. revealed that p38 mitogen-activated protein kinase (MAPK) is activated downstream of Piezo1-mediated Ca^2+^ entry, which causes increased IL-6 secretion (Mitrokhin et al., 2017; Blythe et al., 2019). Together, the role of Piezo1 function in cardiac fibroblasts is illustrated by these recent studies. More research on mechanical signaling and the role of fibroblast-specific Piezo1 in altering cardiac pathophysiology and cardiac remodeling *in vivo* will be required to further define its role in the heart.

#### 4.1.3 Ca^2+^ release-activated Ca^2+^ (CRAC) channels

Recently, the functional calcium release-activated calcium channel protein 1 (Orai1)/stromal interaction molecule 1 (STIM1) channels have been demonstrated by Ca^2+^ imaging measurements in human ventricular fibroblasts (Ross et al., 2017; Mohis et al., 2018; Feng et al., 2019; Humer et al., 2022). Currents through CRAC channels are critically dependent on the correct function of two proteins-Orai1 and STIM1 (Humer et al., 2022). Orai1 is highly expressed in HCFs (Nemoto et al., 2022). A recent study demonstrated that Orai1 expression was upregulated in CFs from failing ventricles, indicating the pivotal role played by Orai1 in HF (Ross et al., 2017). Lee et al. found that fibroblast growth factor 23 (FGF23) (25 ng/mL) increased proliferative and migratory abilities of human atrial fibroblasts. FGF23 (25 ng/mL)-treated cardiac fibroblasts had higher expression levels of Orai1 (Li et al., 2021). A regulatory subunit within the endoplasmic reticulum (ER), STIM1 is partially located inside the ER and able to sense the Ca^2+^ concentration within (Parks et al., 2016; Čendula et al., 2021). STIM1 activates Orai1 to allow Ca^2+^ entry into the cells (Liou et al., 2005; Roos et al., 2005; Čendula et al., 2021). Depletion of Ca^2+^ stores induces a conformational change, which allows STIM after its oligomerization to interact with Orai (Liou et al., 2005; Muik et al., 2011; Trebak et al., 2013). Orai proteins show four transmembrane domains and form hexameric channels in the plasma membrane, which is highly selective for Ca^2+^ (Covington et al., 2010; Muik et al., 2011). These channels are opened after interaction with active STIM proteins, which allows the inflow of Ca^2+^ from the extracellular environment (Čendula et al., 2021). Three genes that encode Orai proteins (Orai1–3) and two encoding STIM proteins (STIM1-2) are currently known (Ruhle and Trebak, 2013; Wen et al., 2016). All three Orai proteins can be activated by STIM1 when Ca^2+^ is low inside the ER, but they differ at key sites that interact with STIM1 (Ruhle and Trebak, 2013; Humer et al., 2022). STIM2 is more sensitive to Ca^2+^ but is weaker in activating Orai (Humer et al., 2022). Cardiac-specific deletion of Orai3 leads to severe dilated cardiomyopathy and heart failure in mice (Gammons et al., 2021). These results suggest that CRAC channels play a role in fibroblast Ca^2+^ signaling, and may therefore contribute to cardiac remodeling under pathological conditions. Non-etheless, further investigations using the fibroblast-specific deletion of Orai/STIM proteins may help to clarify the pathophysiological roles of CRAC channel in the heart (Feng et al., 2019).

#### 4.1.4 Voltage-gated Ca^2+^ channels (VGCCs)

Voltage-gated Ca^2+^ channels (VGCCs) are multisubunit protein complexes embedded in the plasma membranes of a variety of cell types that couple membrane depolarization to intracellular Ca^2+^ elevations and downstream Ca^2+^-dependent signaling processes (Catterall, 1991; Catterall et al., 2005; Felix and Weiss, 2017). The RNA expression of L-type (CaV1.2), T-type (CaV3.3) and R-type (CaV2.3) have been detected in HCFs (Feng et al., 2019; Bae et al., 2020). Bae and colleagues revealed that L-type (CaV1.2) and T-type (CaV3.3) VGCCs were highly expressed in myofibroblasts and that CaV2.3 (R-type) VGCCs showed a low expression in HCFs (Bae et al., 2020). Moreover, MFs can be electrically coupled to myocytes *via* gap junctions and alter the action potential characteristics (Gaudesius et al., 2003; Kohl and Gourdie, 2014; Bae et al., 2020; Boyle et al., 2021; Grune et al., 2021). Whole-cell mode patch-clamp recordings demonstrate the presence of L-type Ca^2+^ (*I*_*Ca*_, L) and T-type Ca^2+^ (*I*_*Ca*_, T) currents in human cardiac myofibroblasts (Bae et al., 2020). To date, 10 genes encoding the pore-forming subunits of VGCCs have been identified and classified into two subfamilies, *viz.*, high voltage-activated (HVA) and low voltage-activated (LVA) channels. L-type (CaV1.2) and R-type (CaV2.3) channels are members of the HVA subfamily, and T-type channel (CaV3.3) is a member of the LVA subfamily (Soong et al., 1993; Klöckner et al., 1999; Lee et al., 1999; Ertel et al., 2000; Catterall et al., 2005; Catterall, 2011; Felix and Weiss, 2017). VGCCs are a vital part of the cellular electrical machinery, as they have the unique ability to translate membrane excitability into cytoplasmic Ca^2+^ changes and thus play key roles in the activation of sarcoplasmic reticulum channels, contractility, proliferation, and apoptosis of cells (Marks, 1997; Catterall, 2011; Pitt et al., 2021; Sánchez et al., 2021). However, to date, the specific effect of VGCCs and their impact on myofibroblast electrophysiology has not been fully described. In addition, the role of calcium in MFs and the electromechanical effects of coupling between MFs and myocytes need further elucidation.

### 4.2 Na^+^ channels and cardiac fibrosis

It has been reported that voltage-gated Na^+^ channels are expressed in CFs. Tetrodotoxin (TTX) sensitivity and Na^+^ current (*I*_*Na*_) are recorded in cultured ventricular fibroblasts (Chatelier et al., 2012). In cultured fibroblasts, TTX-resistant *I*_*Na*_ is found only in differentiated MFs, but not in freshly isolated fibroblasts. In cardiomyocytes, TTX-resistant *I*_*Na*_ presents similar characteristics to *I*_*Na*_, which may be encoded by NaV1.5 α and β1 subunits. The expressions of these two subunits in fibroblasts are significantly lower than those in MFs, so the expression of *I*_*Na*_ in fibroblasts is closely related to its proliferation and differentiation (Chatelier et al., 2012).

Na^+^ current has a unique expression in human atrial MFs. Human atrial fibroblasts show no α-SMA protein expression 7 day before culture but significantly expressed α-SMA protein after 12 day, indicating that fibroblasts differentiate into MFs. The expression of Na^+^ channel subunit Nav1.5 also has the same time course between 7–12 day of culture (Voigt et al., 2012). *I*_*Na*_ can be recorded in all cells 12 day after culture. RT-qPCR, immunofluorescence and pharmacological experiment also show the same results (Dobrev et al., 2012). *I*_*Na*_ is mainly detected by Nav1.5, which is the first time to show *I*_*Na*_ in human atrial MFs (Imoto et al., 2021). As a potential marker of human atrial MFs, *I*_*Na*_ can promote the electrophysiological separation of patients’ atrial fibroblasts and MFs, which may control the transformation of fibroblasts to MFs secreting ECM. Abnormal Ca^2+^ processing is a key determinant of atrial myocyte dysfunction in patients with AF, and abnormal Ca^2+^ entry is considered to be necessary for fibroblasts to proliferate, migrate and differentiate into MFs in the diseased heart. In addition, the reverse mode (Ca^2+^ influx) Na^+^-Ca^2+^ exchanger (NCX) may also be involved in Ca^2+^ signal transduction in MFs (Torres, 2021). MFs *I*_*Na*_ has a larger window current than cardiomyocyte *I*_*Na*_, resulting in greater continuous Na^+^ entry and enhancing Ca^2+^ influx through NCX, which may contribute to cell proliferation (Harada et al., 2012). Moreover, Na^+^ continues to enter fibroblasts, and α-SMA-positive MFs may affect the electrophysiology of cardiomyocytes, and thereby affecting the occurrence of fibrosis through cardiomyocytes, especially both groups are electrically coupled.

### 4.3 K^+^ channels and cardiac fibrosis

**FIGURE 2:**
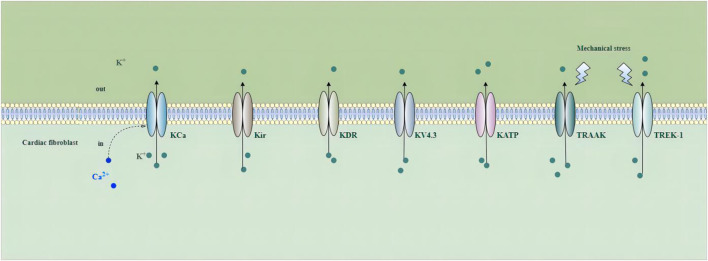
Potassium channels in cardiac fibroblasts. Abbreviations; KCa, Ca^2+^-activated K^+^ channel; Kir, inward rectifier K^+^ channel; KDR, delayed rectifier K^+^ channel; KV4.3, voltage-gated K^+^ channel 4.3; KATP, ATP-sensitive potassium channel; TRAAK, Tandem of P-domains in a weak inward-rectifying K^+^ channel (TWIK)-related arachidonic acid activated K^+^ channel; TREK-1, TWIK-related K^+^ channel 1; See text for details.

Increasing studies have demonstrated that voltage-gated and non-voltage-gated ion channels are important in fibroblast function. The activation of these channels can change intracellular ion levels and subsequent cell activity, and induce the ability of cells to respond and adapt to the environment by promoting the changes in gene expression and cellular remodeling (Zhan and Xia, 2013). Potassium channels play a crucial role in cardiac electrophysiology, and their dysfunction is associated with intracellular signaling, metabolism, remodeling, and arrhythmogenesis in a number of cardiovascular disorders (Grandi et al., 2017). Ca^2+^-activated K^+^ (KCa) channels, inward rectifier K^+^(Kir) channels, delayed rectifier K^+^ (KDR) channels, voltage-gated K^+^ channel 4.3 (Kv4.3) channels, ATP-sensitive potassium (K_ATP_) channels, Tandem of P-domains in a weak inward-rectifying K^+^ (TWIK)-related arachidonic acid activated K^+^ (TRAAK) channels, and TWIK-related K^+^ channel 1 (TREK-1) have been detected in fibroblasts (Choi et al., 2008; Walsh and Zhang, 2008; Benamer et al., 2009; Li et al., 2009; Chen et al., 2016; Pertiwi et al., 2019; Roach and Bradding, 2020) (Figure 2).

In recent years, it has been shown that fibroblasts in the rat heart have the expression of Ca^2+^-activated K^+^ (KCa) channels, including large-conductance KCa (BK), intermediate conductance KCa (IK) and small conductance KCa (SK), the current of three types of SK channels can also be detected using whole-cell patch clamp, and SK channels have four types SK1-SK4 (Choi et al., 2008; Dong et al., 2016; Bae and Lim, 2017). Several types of KCa currents, including large conductance and intermediate conductance (Wang et al., 2006; Li et al., 2009; He et al., 2011), exist in human and rat fibroblasts and play a role in fibroblast proliferation, fibroblast-myocyte coupling and stretching response (Stockbridge and French, 1988).

Studies have shown that HCFs can express Ba^2+^-sensitive inward rectifier K^+^ current (IKir) (likely encoded by Kir2.1 or Kir2.3), 4-aminopyridine (4-AP)-sensitive delayed rectifier K^+^ current (IKDR) (likely encoded by Kv1.5 or Kv1.6), and (4-AP)-sensitive transient-outward K^+^ current (Ito) (likely encoded by Kv4.3) (Li et al., 1998; Li et al., 2009; Sheng et al., 2013). According to Li et al., *IKir* was detected in 24% of HCFs, *Ito* in 15% of HCFs, and *IK*_*DR*_ in 14% of HCFs (Li et al., 2009). Kir channels are members of the inward rectifier K^+^ channel family, with higher conductance during hyperpolarization (favoring K^+^ influx) and decreased conductance during depolarization (Nerbonne and Kass, 2005; Chen et al., 2016). Cardiac KDR channels conduct outward potassium currents during the plateau phase of action potentials and play pivotal roles in cardiac repolarization (Nerbonne and Kass, 2005; Chen et al., 2016). *IKir* activity has been reported to influence MF proliferation and contraction functions during *in vitro* experiments (Chilton et al., 2005). Aguilar et al. conclude that HF induces fibroblast ion-current remodeling with *IKir* upregulation and suggest that fibroblast K^+^-current remodeling is a novel component of AF-related remodeling that might contribute to arrhythmia dynamics (Aguilar et al., 2014). Acute exposure of atrial fibroblasts to hydrogen sulfide facilitates steady-state inactivation of Ito currents and attenuates recovery from inactivation (Sheng et al., 2013). Sheng and colleagues revealed that TGF-β1-induced fibroblast proliferation and differentiation are likely to be accompanied by increased expression of Kv4.3, which could be attenuated by preincubation with hydrogen sulfide, an endogenous gaseous modulator of K^+^ channels (Sheng et al., 2013). In addition, antagonizing Kv4.3 channels might be a potential antifibrotic strategy to prevent the progression of fibrosis, thereby decreasing the substrate for cardiac dysfunctions like AF, cardiomyopathy, and HF. Hyperpolarized membrane potential caused by Kir channel upregulation in fibroblasts can increase the driving force for Ca^2+^ entry *via* mechanisms like store-operated Ca^2+^ entry (SOCE), enhancing intracellular Ca^2+^-dependent processes that include profibrotic factor, cytokine, and collagen secretion, as well as promoting fibroblast proliferation (Clapham, 2007; Penna and Stutzin, 2015; Qi et al., 2015).

Another significant K^+^ conductance in fibroblasts is the ATP-sensitive potassium (K_ATP_) channel. The activation of K_ATP_ in mouse cardiac fibroblasts can increase cell proliferation, reduce IL-6 secretion (Benamer et al., 2009), and inhibit fibroblast differentiation. MI can induce the expression of functional K_ATP_ in fibroblasts in scar and boundary areas, which may affect the electrophysiological characteristics of myocytes. In mouse and rat hearts, K_ATP_ channel has been proved to be expressed in cardiomyocytes and myocardial fibroblasts, but K_ATP_ may not play a role under physiological conditions (Nichols, 2016). However, K_ATP_ current can be detected in fibroblasts isolated from infarct scar and boundary areas, which indicates that K_ATP_ channel may be active only under pathological conditions. The function of the fibroblast K_ATP_ channel contributes to cardiac remodeling and electrophysiology in scar boundary areas after MI (Huang et al., 2013; Kobara et al., 2022), and may reduce the depolarization effect of myocardial fibroblasts on cardiomyocytes. When rat fibroblasts transdifferentiate into MF phenotypes, the expression of K_ATP_ increases, which is related to increased α-SMA expression. Patch-clamp results showed K_ATP_ current and α-SMA expression in fibroblasts cultured for several days, suggesting that K_ATP_ is involved in a reaction mechanism of pathological signal transduction in fibroblasts.

Recently, the role of two-pore-domain K^+^ (K2P) channels and their potential have been of increasing interest. K2P channels, a superfamily of potassium channels, have the topology of four transmembrane–spanning domains (M1–M4) and two pore-forming domains in tandem (Fink et al., 1996; Kamatham et al., 2019). K2P channels serve an essential role in cardiac function. K2P channels mediate background potassium currents that stabilize the resting membrane potential and contribute to the repolarization of action potentials; inhibition or genetic inactivation of K2P currents results in action potential prolongation (Goldstein et al., 2001; Enyedi and Czirják, 2010; Schmidt et al., 2014a; Bodnár et al., 2015; Decher et al., 2015; Schmidt et al., 2015). To date, of the fifteen different types of K2P channels, only Tandem of P-domains in a weak inward-rectifying K^+^ channel 1 (TWIK-1), TWIK-related K^+^ channel 1 (TREK-1), TWIK-related K^+^ channel 2 (TREK-2), TWIK-related arachidonic acid activated K^+^ channel (TRAAK), TWIK-related acid-sensitive K^+^ channel 1 (TASK-1), TWIK-related acid-sensitive K^+^ channel 2 (TASK-2), TWIK-related acid-sensitive K^+^ channel 3 (TASK-3), Tandem pore domain halothane inhibited K^+^ channel 1 (THIK-1), and TWIK-related alkaline pH-activated K^+^ channel 2 (TALK-2) have been described as expressed and functional in the human heart (Fink et al., 1998; Medhurst et al., 2001; Barth et al., 2005; Ellinghaus et al., 2005; Gaborit et al., 2009; Limberg et al., 2011; Staudacher et al., 2011; Kisselbach et al., 2014; Rahm et al., 2014; Christensen et al., 2016). Two K2P subfamily members, TASK and TREK channels, are highly expressed in human cardiac tissue and implicated in cardiac arrhythmogenesis (Gurney and Manoury, 2009; Schmidt et al., 2017). However, until recently, only TREK-1 and TRAAK have been reported as being functionally expressed in CFs (Abraham et al., 2018; Zhao et al., 2021). Cellular swelling or stretch induced by pathology could alter the normal function of K2P family channels such as TREK-1, TREK-2, and TRAAK, causing cellular excitability caused by stretch-activated signaling (Wiedmann et al., 2016; Khoubza et al., 2021). In addition, TREK-1 is intrinsically mechanosensitive, and patch clamp recordings of TREK-1 channels reconstituted in liposomes demonstrated that it could activate in response to membrane tension in the absence of other proteins and cytoplasmic tethers (Patel et al., 1998; Berrier et al., 2013; Brohawn et al., 2014b). TRAAK and TREK-1 have similar properties in regulating cellular excitability under conditions of mechanical disturbance or metabolic stress (Brohawn et al., 2014a; Brohawn et al., 2014b; Zhao et al., 2021). Non-etheless, the responses of TREK-1 and TRAAK to mechanical stimuli are quite different. In the *in vitro* recombination pathway, TRAAK has a lower threshold to respond to mechanical stimulation than TREK-1 (Peyronnet et al., 2016). Research shows that TRAAK messenger RNA (mRNA) expression was downregulated in hypoxia-induced rat cardiac fibroblasts and TAC-induced mouse hearts (Zhao et al., 2021). Zhao et al. (2021) suggest that cardiac fibroblast TREK-1 plays a crucial role in the pathogenesis of pressure overload-induced cardiac dysfunction. In addition, unlike the low open probability of TRAAK in the absence of membranous tension, TREK-1 was robustly activated after increasing mechanical force *via* conformational changes in the gating that favor channel opening. While TREK-1 functionality has predominantly been studied in the nervous system, this potassium channel has also been shown to be expressed and functional within the fibroblasts of mouse hearts (Abraham et al., 2018; Stewart and Turner, 2021). It is also well established that cardiac TREK-1 dysregulation promotes the development of AF, ventricular arrhythmias, and HF (Schmidt et al., 2014a; Schmidt et al., 2014b; Kisselbach et al., 2014; Lugenbiel et al., 2017; Schmidt et al., 2017; Abraham et al., 2018; Kamatham et al., 2019; Wiedmann et al., 2021). In addition, TREK-1 is intrinsically mechanosensitive, and patch clamp recordings of TREK-1 channels reconstituted in liposomes demonstrated that it could activate in response to membrane tension in the absence of other proteins and cytoplasmic tethers (Patel et al., 1998; Berrier et al., 2013; Brohawn et al., 2014b). While cardiomyocyte-specific deletion of TREK-1 in response to *in vivo* pressure overload resulted in cardiac dysfunction, TREK-1 deletion in fibroblasts prevented deterioration in cardiac function (Abraham et al., 2018). Abraham et al. suggest that cardiac fibroblast TREK-1 plays a key role in the pathogenesis of pressure overload-induced cardiac dysfunction (Abraham et al., 2018). TREK-1 is an important regulator of cardiac hypertrophy, diastolic function, and fibrosis in mice, and its deletion significantly reduces cardiac fibrosis in mice after aortic coarctation (Kamatham et al., 2019). To summarize, cardiac TREK-1 appears to play a homeostatic role in electrical signaling, but it may also influence pathological cardiac remodelling.

## 5 Conclusion

In recent years, ion channels have been considered key regulators of cardiac fibroblast functions, which are important for fibrotic cardiac remodeling at the fibroblast level, including cell proliferation, MF differentiation, ECM turnover and paracrine signal transduction. The complex regulation of ion channel signal transduction is gradually being revealed. Increasing evidence shows that ion channels in fibroblasts are involved in the pathophysiological consequences of cardiac infarction, such as myocardial hypertrophy, fibrosis and angiogenesis after ischemia. Therefore, strengthening the understanding of the role and mechanism of ion channels in fibroblasts will help to identify new therapeutic targets for drug development, so as to reduce the impact of fibrotic remodeling on cardiac function in heart diseases.
